# Derivation of prediction error variance for non-genotyped individuals in genomic selection

**DOI:** 10.3389/fgene.2026.1792190

**Published:** 2026-06-03

**Authors:** Vinícius Silva Junqueira, Marcos Jun-Iti Yokoo, Fernando Flores Cardoso

**Affiliations:** 1 Bayer R&D, Bayer Crop Science, Uberlândia, Brazil; 2 Embrapa Southeastern Livestock, São Carlos, Brazil; 3 Embrapa South Livestock, Bagé, Brazil

**Keywords:** budget constrained, matrix decomposition, mixed model equations, prediction uncertainty, schur complement

## Abstract

Genomic selection has transformed plant and animal breeding by enabling accurate prediction of genetic merit using DNA markers; however, comprehensive genotyping of all selection candidates remains economically prohibitive for most breeding programs. While breeding programs must decide which subset of individuals to genotype within budget constraints, current approaches rely primarily on experience-based decisions rather than quantitative frameworks. We present explicit mathematical derivations for prediction error variance (PEV) in non-genotyped individuals under mixed model equations, providing a theoretical foundation for evaluating genotyping strategies prospectively. The approach derives PEV expressions for non-genotyped selection candidates under different relationship matrix structures, including pedigree-based, genomic, and hybrid single-step methodologies that combine both information sources. The derivations accommodate complex breeding program structures with historical training populations containing both genotypes and phenotypes alongside contemporary selection candidates with only pedigree information. Using Schur complement methods applied to partitioned mixed model equations, the framework enables calculation of prediction uncertainty without requiring actual phenotypic data from selection candidates. The expressions simplify under different information scenarios, from cases with complete phenotypic data to situations where only relationship information is available. The method was validated through simulations across six scenarios with populations ranging from 180 to 15,500 individuals, confirming numerical equivalence with direct matrix inversion while demonstrating computational and memory advantages that increase with population size. Although genomic relationship matrix operations dominate the complexity, matrix decomposition techniques, including Cholesky factorization and APY methodology, can improve efficiency. The mathematical framework provides quantitative tools for transitioning from experience-based to mathematically-informed genotyping decisions, with applications extending to any field requiring prospective quantification of prediction uncertainty under resource constraints.

## 1 Introduction

Genomic selection has revolutionized plant and animal breeding by enabling the prediction of genetic merit using DNA markers (Meuwissen et al., 2001), fundamentally changing how breeding programs allocate resources and make selection decisions. Since its introduction 2 decades ago, genomic selection has delivered genetic gains across diverse species, from cattle (Garrick, 2011; Wiggans et al., 2017; Guinan et al., 2023) and pigs (Sharif-Islam et al., 2024) to wheat (He et al., 2016) and maize (Crossa et al., 2017), by allowing breeders to identify superior individuals earlier in their development and with higher accuracy than traditional phenotypic selection methods. However, despite dramatic reductions in genotyping costs, comprehensive genotyping of all selection candidates remains economically prohibitive for some breeding programs, creating a fundamental challenge: determining which individuals should be genotyped to maximize genetic improvement within budgetary constraints. This challenge is especially acute in commercial breeding programs, which generate thousands of selection candidates annually, while genotyping budgets typically cover only a small fraction of them. For example, in animal breeding (e.g., cattle, poultry, and pigs), all individuals have pedigree-based breeding values regardless of phenotyping status, allowing for initial pre-selection based on performance. Pre-selected individuals are subsequently phenotyped, but genotyping strategies differ by species and company budget. While large poultry and pig breeding companies may typically genotype a large number of pre-selected individuals, smaller companies and all cattle breeding programs cannot afford this approach. Consequently, many individuals that ultimately contribute phenotypes to the training set remain ungenotyped. In these situations, prioritization rules must be employed to define which individuals to genotype under budget constraints.

Practical examples highlight the extent of this resource constraint. In Brazilian beef cattle breeding, the PROMEBO Angus program has genotyped only 9.4% of phenotyped animals, while the Brangus + program has genotyped 25.5%. In contrast, poultry breeding programs typically genotype all layers but only a selected subset of males from elite families (personal communication). Similar resource allocation decisions arise with emerging sequencing technologies, where programs must determine which individuals to sequence at higher densities or depths. Across all these scenarios, the question remains: which individuals should be prioritized for genotyping to maximize their contribution to prediction accuracy and genetic gain under budget constraints?

Genotyping decisions directly impact selection accuracy and overall operational efficiency, with suboptimal choices reducing genetic progress and return on investment in genomic technologies (Lopez-Cruz and De Los Campos, 2021). Traditional approaches to this problem have relied heavily on breeder experience or simple rules of thumb, such as genotyping a fixed proportion of individuals from each family (Isidro et al., 2015). While these methods have proven workable, they lack the mathematical foundation needed to quantitatively evaluate competing genotyping strategies or to balance objectives such as maximizing prediction accuracy while efficiently utilizing limited resources across complex breeding population structures.

The prediction error variance (PEV) from mixed model theory provides a natural mathematical framework for quantifying prediction uncertainty, as it captures the expected accuracy of genetic predictions (Henderson et al., 1984) before genotyping decisions are implemented. PEV represents the uncertainty associated with predicted breeding values, with lower values indicating higher prediction accuracy (Misztal and Wiggans, 1988). The prediction accuracy is proportional to the amount of information used to compute breeding values, with sources of information being pedigree, phenotypes, and genotypes.

Modern breeding programs often involve complex population structures that encompass historical training populations with both genotypic and phenotypic data, alongside contemporary young candidates that may only have pedigree information. This configuration enables the construction of joint relationship matrices that integrate genomic and pedigree information through single-step methodologies (Aguilar et al., 2010; Christensen et al., 2012). The mathematical framework of this study derives explicit PEV expressions under different relationship matrix structures, providing the theoretical foundation for the quantitative evaluation of genotyping strategies. The practical value of these expressions extends to fundamental questions about breeding program design, including training population composition (Rincent et al., 2012; Akdemir and Isidro-Sánchez, 2019), genetic diversity maintenance, and the integration of phenotypic and genomic data collection strategies.

Although several studies have previously addressed training set design for genomic selection, they assume genotypic information is available for all individuals (Rincent et al., 2012; Akdemir and Isidro-Sánchez, 2019). However, in many breeding programs, phenotyped individuals in the training set lack genotypes due to budget constraints. To our knowledge, no previous study has developed a quantitative method to guide the selection of which individuals to genotype under such resource limitations. Here, we develop explicit mathematical expressions for PEV in non-genotyped individuals that will eventually contribute phenotypes to the training population.

## 2 Methods

Consider a breeding program using genomic selection where candidates are first preselected based on pedigree-based breeding values. These individuals are then phenotyped and added to the training population. However, budget constraints typically prevent genotyping all preselected candidates, requiring the breeding program to prioritize which individuals will be genotyped.

This study addresses this optimization problem by deriving PEV equations for ungenotyped individuals. These equations provide a quantitative basis for prioritizing genotyping decisions and can be integrated into optimization algorithms such as differential evolution, integer programming, or simulated annealing with cost-related constraints, enabling breeders to maximize genetic gains within budget limitations. The following sections establish the mixed model equations, derive explicit expressions for prediction error variance in non-genotyped individuals, and examine how PEV calculations simplify under different information scenarios. While the practical implementation of these equations in an optimization algorithm will be presented in a future publication, we briefly describe the rationale for such implementation in this paper.

### 2.1 Linear mixed model framework

Consider the standard mixed model equation (MME) for genetic evaluation:

y=Xβ+Zu+e

where y is the vector of phenotypic observations, X is the design matrix for fixed effects β , Z is the design matrix relating observations to individuals, u is the vector of random additive effects and e is the vector of residual effects.

The variance structure is defined as:

$$\text{Var}\left(\begin{array}{c} u\\ e \end{array}\right)=\left(\begin{array}{cc} G & 0\\ 0 & R \end{array}\right)$$

where $G=H\sigma_{u}$ , $\sigma_{u}$ is the variance of additive effects, H the joint relationship matrix that combines pedigree and genomic information (Aguilar et al., 2010; Christensen and Lund, 2010). The residual term of the equations is $R=I\sigma_{e}$ , $\sigma_{e}$ is the residual variance.

### 2.2 Partitioned system for training and young individuals

As our objective is to decide which young individuals should be genotyped, partitioning the coefficient matrix provides a convenient way to simplify the derivations for this group, while still accounting for information from the entire training population. Accordingly, individuals are partitioned into two groups: a training set (with historical phenotypic and genotypic data) and a young set (available only through pedigree). Initially, a general matrix G can be partitioned into training (t) and young (y) components, along with their cross-components, as follows:

$$G=\left[\begin{array}{cc} G_{tt} & G_{ty}\\ G_{yt} & G_{yy} \end{array}\right]$$

where $G_{tt}$ is the genetic covariance matrix among training individuals, $G_{yy}$ among young candidates, and $G_{ty}=G_{yt}^{⊤}$ the cross-covariance between the two groups.

The inverse of the partitioned matrix G can be written as:

$$G^{-1}=\left[\begin{array}{cc} G^{tt} & G^{ty}\\ G^{yt} & G^{yy} \end{array}\right]$$

The following presents the partitioned form of the MME, with explicit separation of training and young candidate blocks:

$$\begin{array}{ll} & \left[\begin{array}{ccc} X^{T}R^{-1}X & X^{T}R^{-1}Z_{t} & X^{T}R^{-1}Z_{y}\\ Z_{t}^{T}R^{-1}X & Z_{t}^{T}R^{-1}Z_{t}+G^{tt} & Z_{t}^{T}R^{-1}Z_{y}+G^{ty}\\ Z_{y}^{T}R^{-1}X & Z_{y}^{T}R^{-1}Z_{t}+G^{yt} & Z_{y}^{T}R^{-1}Z_{y}+G^{yy} \end{array}\right]\times \\ & \left[\begin{array}{c} \hat{\beta }\\ {\hat{u}}_{t}\\ {\hat{u}}_{y} \end{array}\right]=\left[\begin{array}{c} X^{T}R^{-1}y\\ Z_{t}^{T}R^{-1}y\\ Z_{y}^{T}R^{-1}y \end{array}\right] \end{array}$$

However, as the young candidates do not have phenotypes, the MME can be simplified as follows:

$$\left[\begin{array}{ccc} X^{T}R^{-1}X & X^{T}R^{-1}Z_{t} & 0_{n_{t}}\\ Z_{t}^{T}R^{-1}X & Z_{t}^{T}R^{-1}Z_{t}+G^{tt} & G^{ty}\\ 0_{n_{y}} & G^{yt} & G^{yy} \end{array}\right]\left[\begin{array}{c} \hat{\beta }\\ {\hat{u}}_{t}\\ {\hat{u}}_{y} \end{array}\right]=\left[\begin{array}{c} X^{T}R^{-1}y\\ Z_{t}^{T}R^{-1}y_{t}\\ 0_{n_{y}} \end{array}\right]$$

An important structural insight of the partitioned mixed model equations is that young individuals without phenotypic records contribute nothing to the cross-product terms $Z_{y}^{⊤}R^{-1}Z_{y}$ , $Z_{t}^{⊤}R^{-1}Z_{y}$ , and $X^{⊤}R^{-1}Z_{y}$ in the coefficient matrix. Consequently, the corresponding block of the right-hand side reduces to a null vector with the length as the number of unphenotyped individuals $\left(0_{n_{y}}\right)$ .

### 2.3 Prediction error variance

Following Henderson et al. (1984), the prediction error variance is defined as

$$\text{PEV}=\text{Var }\left(u-\hat{u}\right)$$

Expanding the variance of the prediction error:

$$\begin{array}{ll} \text{PEV } & =\text{Var }\left(u-\hat{u}\right)\\ & =\text{Var }\left(u\right)+\text{Var }\left(\hat{u}\right)-2\text{Cov }\left(u,\hat{u}\right) \end{array}$$

Since $\hat{u}$ is the BLUP of u , and using the property that $\text{Cov }\left(u,\hat{u}\right)=\text{Var }\left(\hat{u}\right)$ for best linear unbiased predictors:

$$\begin{array}{ll} \text{PEV } & =\text{Var }\left(u\right)-\text{Var }\left(\hat{u}\right)\\ & =\text{Var }\left(u\right)-\text{Cov }\left(u,\hat{u}\right)\\ & =G-\text{Cov }\left(u,\hat{u}\right)\\ & =C^{-1} \end{array}$$

where C is the coefficient matrix (i.e., left-hand side block) of the mixed model equations and ${\left(C^{-1}\right)}_{uu}$ is the block corresponding to the random effects.

Therefore, the prediction error variance equals:

$$\text{PEV}=C_{uu}^{-1}$$

In practice, the interest lies only in the prediction error variance of the young individuals. Therefore, the direct inversion of the full coefficient matrix would not be necessary. By partitioning the MME and applying the Schur complement (Ouellette, 1981) to the $u_{y}$ -block, it is possible to obtain the relevant block of the inverse directly. This approach not only simplifies the algebra but also has a clear interpretation: the Schur complement represents the information available for the young individuals after accounting for the training population and fixed effects. Thus, applying the Schur complement to the $u_{y}$ -block, the PEV of $u_{y}$ can be expressed as

$$\begin{array}{ll} B & =\left[\begin{array}{cc} X^{⊤}R^{-1}X & X^{⊤}R^{-1}Z_{t}\\ Z_{t}^{⊤}R^{-1}X & Z_{t}^{⊤}R^{-1}Z_{t}+G^{tt} \end{array}\right],\\ E & =\left[\begin{array}{c} 0\\ G^{ty} \end{array}\right],\\ F & =G^{yy}. \end{array}$$

Then the full coefficient matrix can be written as

$$D=\left[\begin{array}{cc} B & E\\ E^{⊤} & F \end{array}\right]$$

Then, following the rules for the inverse of Schur Complement, the block corresponding to the $u_{y}$ parameters is given by the following block-inverse formula:

$$S^{-1}={\left(F-E^{⊤}B^{-1}E\right)}^{-1}$$

where $S=F-E^{⊤}B^{-1}E$ is the Schur complement of B -block in D .

Therefore:

$$\text{PEV }\left(u_{y}\right)=S^{-1}$$

This Schur complement captures the conditional information about the young individuals after accounting for the training population and fixed effects through the term $E^{⊤}B^{-1}E$ .

### 2.4 PEV for ungenotyped individuals

Using the Schur complement of B in the partitioned system and substituting the blocks for young and training individuals, the information matrix for $u_{y}$ is

$$S=G^{yy}-G^{yt}B^{22}G^{ty},$$

where $B^{22}$ denotes the $\left(u_{t},u_{t}\right)$ block of $B^{-1}$ . Using the block matrix inversion formula (see Appendix 1 for derivation), $B^{22}$ equals:

$$B^{22}={\left[\left(Z_{t}^{⊤}R^{-1}Z_{t}+G^{tt}\right)-Z_{t}^{⊤}R^{-1}X{\left(X^{⊤}R^{-1}X\right)}^{-1}X^{⊤}R^{-1}Z_{t}\right]}^{-1}$$

Hence, the prediction error variance of the young individuals is

$$\text{PEV }\left(u_{y}\right)=S^{-1}={\left[G^{yy}-G^{yt}B^{22}G^{ty}\right]}^{-1}$$ (1)

#### 2.4.1 Simplified PEV expressions

When phenotypic information from the training population is not utilized or is limited, simplified versions of Equation 1 can be derived. Three such cases are presented below, corresponding to scenarios where X , Z , and their cross-products from MME can be disregarded.

##### 2.4.1.1 Case 1: no fixed effects

In many practical situations, fixed effect terms may be omitted from the model or their contribution may be negligible. When fixed effects are omitted, the X -related blocks vanish from the mixed model equations. The coefficient matrix for ${\left[u_{t}^{⊤},\;u_{y}^{⊤}\right]}^{⊤}$ reduces to

$$\;B\;=\;Z_{t}^{⊤}R^{-1}Z_{t}\;+\;G^{tt}$$

Assuming B is nonsingular, the Schur complement of B in D is

$$S\;=\;F-E^{⊤}B^{-1}E\;=\;G^{yy}-G^{yt}\;{\left(Z_{t}^{⊤}R^{-1}Z_{t}+G^{tt}\right)}^{-1}\;G^{ty}.$$

Using Equation 1 as reference, the PEV for the young candidates is

$$\text{PEV }\left(u_{y}\right)\;=S^{-1}\;={\left[G^{yy}-G^{yt}\;{\left(Z_{t}^{⊤}R^{-1}Z_{t}+G^{tt}\right)}^{-1}\;G^{ty}\right]}^{-1}\;$$

##### 2.4.1.2 Case 2: no phenotypic data

When phenotypic information is ignored, $Z_{t}^{⊤}R^{-1}Z_{t}$ , $Z_{t}^{⊤}R^{-1}Z_{y}$ , $Z_{t}^{⊤}R^{-1}X$ , and $X^{⊤}R^{-1}X$ of B are zero. In this limiting case the X and Z -related blocks disappear from the MME, and only the relationship block remains. Consequently, B reduces to $G^{tt}$ , and $B^{22}$ simplifies to:

$$B^{22}={\left(G^{tt}\right)}^{-1}$$

Substituting this into the PEV expression:

$$\text{PEV }\left(u_{y}\right)={\left[G^{yy}-G^{yt}{\left(G^{tt}\right)}^{-1}G^{ty}\right]}^{-1}$$

##### 2.4.1.3 Case 3: weak phenotypic information

When phenotypic information is weak relative to the genetic information (i.e., $\text{tr}\left(Z_{t}^{⊤}R^{-1}Z_{t}\right)/\text{tr}\left(G^{tt}\right)\ll 1$ ) (Misztal and Wiggans, 1988; Meyer, 1989), the matrix $B^{22}$ can be approximated as

$$\text{PEV}\left(u_{y}\right)\approx {\left[G^{yy}-G^{yt}{\left(G^{tt}\right)}^{-1}G^{ty}\right]}^{-1}$$

This approximation applies when: (i) traits have very low heritability, or (ii) the training population has substantial missing phenotypes.

These simplified cases illustrate successive reductions in information available to the mixed model equations, with computational complexity decreasing as phenotypic data and fixed effects are removed.

##### 2.4.1.4 Estimation of variance components

Across all three cases, the genetic covariance matrix requires both a relationship matrix and an estimate of the additive and residual variances. The relationship matrix H can be constructed using pedigree information alone (Henderson, 1976), genomic markers (VanRaden, 2008), or a combination of both through single-step methods (Aguilar et al., 2010; Christensen et al., 2012). The variance components $\sigma_{u}^{2}$ and $\sigma_{e}^{2}$ are typically estimated using restricted maximum likelihood (REML) (Patterson and Thompson, 1971) or Bayesian methods (Gianola et al., 2009) from available phenotypic and relationship data in the training population.

#### 2.5 Simulation design for validation

To evaluate the similarity between the equations derived in this study and the direct inversion of the coefficient matrix of the MME, we designed a simulation scenario with six problem sizes to assess computational scalability. The simulation created populations ranging from 180 to 15,500 individuals, with varying proportions of genotyped individuals to reflect realistic breeding program structures (Table 1). Each population consisted of founders and their progeny, with young selection candidates representing the most recent generation. Founders ranged from 30 to 500 individuals across problem sizes, with 33% designated as sires and the remaining as dams. Progeny generations (150–15,000 individuals) were simulated with diverse family structures: 25% as full-sibs (sharing both parents), 35% as paternal half-sibs (sharing only sires), 25% as maternal half-sibs (sharing only dams), and 15% with only one known parent. Pedigree records were constructed by recording the sire and dam assignments for each progeny individual. Founders were assigned as unrelated base animals (i.e., with unknown parents). For each progeny individual, a sire was randomly sampled from the pool of male founders and a dam from the pool of female founders, according to the family structure proportions described above. Full-sibs shared both the same sire and dam, paternal half-sibs shared only the sire, and maternal half-sibs shared only the dam. Individuals with only one known parent had the other parent recorded as missing. The pedigree-based relationship matrix (A) was then constructed using Henderson, (1976) method, and the $A_{22}^{-1}$ submatrix corresponding to genotyped individuals was extracted for use in the single-step blending procedure. Young selection candidates (30–3,000 individuals) represented the final cohort and had only pedigree information available, whereas training populations (100–8,000 genotyped individuals) included founders and selected progeny characterized by genotypes, pedigree records, and phenotypes. Genotypes were simulated for 5,000 to 20,000 bi-allelic SNP markers with allele frequencies drawn from a uniform distribution. Marker genotypes for founders were randomly sampled assuming the Hardy-Weinberg equilibrium, while progeny genotypes followed Mendelian inheritance patterns based on parental genotypes when available. The genomic relationship matrix was constructed using VanRaden’s method (VanRaden, 2008). Single-step GBLUP (Aguilar et al., 2010) was implemented by blending genomic matrix with the pedigree-based relationship matrix using weights of 0.95 and 0.05, respectively, to construct the $H^{-1}$ matrix. Variance components were set to $\sigma_{e}^{2}=1.0$ and $\sigma_{u}^{2}=0.5$ , yielding a heritability of 0.33. Each scenario was replicated five times with different random seeds to ensure robust timing estimates and numerical precision assessments. Further details on the data are available in the R code available on GitHub. Peak memory usage was measured for each method using R’s garbage collection diagnostics, recording memory allocation before and after each computation to quantify the memory footprint of each approach. To validate the derived expressions, we compared the direct inversion of the full coefficient matrix of the MME versus the Schur complement approach derived in this study. All analyses were performed on an Apple M2 processor with 8 cores and 16 GB of RAM, running macOS 26.4.1 and R version 4.5.2.

**TABLE 1:** Population structure for each simulation scenario.

Component	S1	S2	S3	S4	S5	S6
Founders	30	50	100	150	300	500
Progeny	150	500	1,000	2,000	8,000	15,000
Training (genotyped)	100	300	600	1,200	5,000	8,000
Young candidates	30	100	200	400	1,500	3,000
Total individuals	180	550	1,100	2,150	8,300	15,500
SNP markers	5,000	8,000	10,000	12,000	15,000	20,000

Values represent the number of individuals in each category.

## 3 Results and discussion

The approach presented in this study applies to breeding programs practicing genomic selection that must decide which individuals to genotype under budget constraints. While genotyping all selection candidates would be ideal, this remains economically unfeasible for many species and companies, as evidenced by animal breeding programs that routinely genotype only a fraction of top-performance individuals. Genotyping strategies in commercial breeding programs are often closely guarded as part of competitive business plans. Although animal and crop commercial breeding programs continue to allocate increasing budgets to genotyping, strategic resource allocation decisions remain a key aspect when evaluated from a return on investment perspective, particularly as breeding programs scale and must balance genotyping comprehensiveness against other operational priorities.

Although several studies have addressed training set optimization using quantitative genetics approaches (Akdemir et al., 2015; Fernández-González et al., 2023), to our knowledge, this is the first study to address genotyping decisions under resource constraints in practical breeding programs. The derivations provide quantitative genetic tools to address these resource-allocation challenges by placing the genetic evaluation model at the center of genotyping decisions. The method exploits genetic relationships between training and ungenotyped individuals to calculate PEV using only relationship matrices and variance components, thereby eliminating the need for actual phenotypic data from ungenotyped individuals. The explicit PEV expressions enable prospective evaluation of prediction uncertainty under different genotyping scenarios, establishing the mathematical foundation for transitioning from experience-based to quantitatively informed genotyping decisions.

Genotyping decisions directly affect selection accuracy and genetic gain (Hayes et al., 2009; Rocha et al., 2025), with important implications for the long-term sustainability and effectiveness of genomic selection programs. The prospective evaluation framework developed here naturally extends beyond traditional genotyping to emerging sequencing technologies (Sthapit et al., 2025), where breeding programs face analogous resource allocation decisions about optimal sequencing depth and coverage. From a crop breeding lens, the approach is valuable in modern crop breeding programs that have adopted schemes with shorter recombination cycles (Gaynor et al., 2017; Gorjanc et al., 2018), where outbred parents have become the norm rather than traditional inbred lines. As newer genotyping platforms become increasingly cost-effective, the method can inform strategic decisions about which individuals to sequence at higher densities, thereby improving imputation accuracy across selection candidates and optimizing resource allocation within breeding pipelines.

**FIGURE 1:**
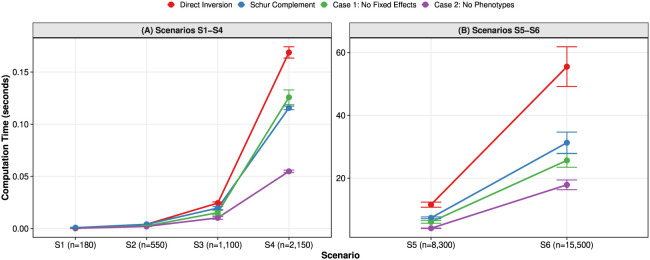
Computation time (in seconds) for prediction error variance (PEV) estimation across scenarios of increasing population size. **(A)** shows scenarios S1–S4 and **(B)** shows scenarios S5–S6, with independent y-axis scales to visualize differences across all methods. Results are shown for four methods: direct inversion of the full mixed model equations (MME) coefficient matrix, the Schur complement method derived in this study, and simplified Cases 1 and 2 (Case 1: no fixed effects; Case 2: no phenotypic data). Error bars represent standard deviations across five replicates.

**FIGURE 2:**
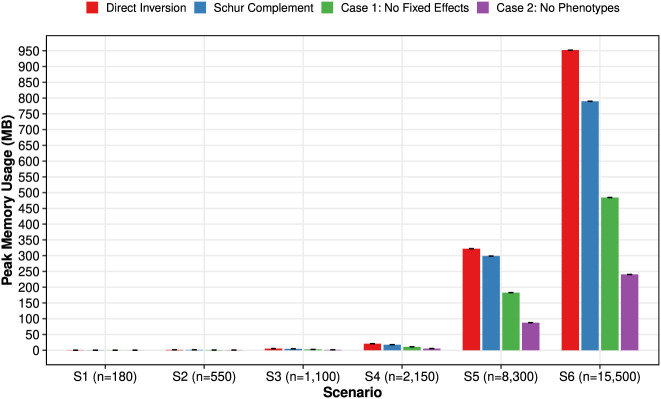
Peak memory usage (in MB) for prediction error variance (PEV) estimation across scenarios of increasing population size. Results are shown for four methods: direct inversion of the full MME coefficient matrix, the Schur complement method, and simplified Cases 1 and 2.

As expected, PEV estimated using Equation 1 in the simulations produced results numerically identical to those obtained by direct inversion of the MME across all six scenarios (Table 1), confirming the mathematical equivalence of the derivations. Figure 1 presents the computational performance of all methods evaluated across scenarios ranging from 180 to 15,500 individuals. The Schur complement method consistently outperformed direct inversion of the full MME coefficient matrix, with the computational advantage increasing at larger population sizes from approximately 20% reduction in computation time at S3 (n=1,100) to 44% at S6 ( n=15,500 ; 55.5 versus 31.3 s). Among the simplified cases, progressive removal of model components yielded corresponding reductions in computation time. Case 1 (no fixed effects) reduced computation by eliminating the absorption of fixed effect equations, and Case 2 (no phenotypic data) further reduced the burden by removing all data-dependent cross-product terms. At the largest scale (S6), Case 2 required only 17.9 s compared to 55.5 s for direct inversion. In addition to computation time, peak memory usage was monitored across all scenarios (Figure 2). The Schur complement method required less memory than direct MME inversion across all scenarios, with the difference becoming more pronounced at larger population sizes. At S6, direct inversion required approximately 952 MB compared to 790 MB for the Schur complement, while Case 2 required only 240 MB. Furthermore, the integration of dimensionality reduction techniques such as the Algorithm for Proven and Young (APY) (Misztal et al., 2015) or eigen decomposition methods could yield additional improvements in computational efficiency, particularly for problems involving dense genomic relationship matrices where matrix operations, especially inversion (Junqueira et al., 2022), dominate the computational burden.

### 3.1 Application to breeding programs

Although practical implementation was beyond the scope of this study, we briefly outline the implementation of the method. A comprehensive evaluation of the method’s performance will be presented in a subsequent publication.

Prediction error variance has been well established since the 1970s (Henderson, 1975; Henderson, 1976), and the formulas derived in this study follow this same theoretical foundation. Estimating PEV for ungenotyped and unphenotyped individuals is straightforward as it is a function only of parents’ PEV. The benefits of applying the equations presented in this study come when integrating it with simulating progeny genotypes based on parental genomic information, which is a process extensively documented in the literature (Bernardo, 2014; Mohammadi et al., 2015; Pook et al., 2020; Gaynor et al., 2021). These simulated genotypes can then be used to construct the relationship matrices $G_{yt}$ and $G_{yy}$ to capture Mendelian sampling variation and genomic similarity to the training population, moving beyond simple pedigree-based relationships. These simulated genotypes are then incorporated into Equation 1 to calculate PEV values for virtual progeny. Although the actual genotypes of future progeny remain unknown, simulating multiple potential progeny from each mating provides insight into the range of genotypes that could arise. Consequently, the average PEV across simulated progeny offers a reliable approximation of expected prediction uncertainty for offspring from specific matings, enabling prospective evaluation of genotyping strategies before progeny are generated.

The selection of which individuals to genotype can be formulated as a combinatorial linear or non-linear optimization problem (Chopard and Tomassini, 2018) with an appropriate objective function subject to budgetary and operational constraints. Several alternative objective functions can be formulated for optimizing genotyping decisions. For example, one straightforward approach is to minimize the average PEV across all ungenotyped individuals estimated using Equation 1. Although computationally feasible, this objective may not represent the optimal strategy for practical breeding programs, as it tends to prioritize genotyping individuals most closely related to the training population. While such individuals benefit from lower prediction uncertainty, this strategy creates a genetically narrow training population that inadequately represents diversity across family structures. This leads to biased predictions that favor closely related individuals at the expense of prediction accuracy for underrepresented families, ultimately compromising both short-term selection decisions and long-term genetic progress. This concern is particularly relevant for breeding programs with larger effective population sizes (Ne), where greater genetic diversity and more complex family structures necessitate broader representation across genetic backgrounds in the training population (Pocrnic et al., 2016). Alternative objective functions could address these limitations by incorporating diversity constraints, such as penalizing excessive relatedness among selected individuals or explicitly balancing prediction error variance against maintenance of genetic diversity within the genotyped subset. Such multi-objective optimization problems can be solved using various algorithms, including differential evolution, integer programming, or simulated annealing.

While this study focuses on genotyping decisions, the framework extends naturally to other resource allocation problems in breeding programs, as Pocrnic et al. (Pocrnic et al., 2022) have discussed. Beyond core subset optimization for APY, Pocrnic et al. (2022) identified several applications following the same underlying rationale. These include prioritizing individuals for high-density genotyping or sequencing, designing selective phenotyping strategies where phenotyping resources are limited, and managing genetic diversity in genebanks. The present study provides an analogous framework for the upstream genotyping decision problem. Whether the objective is selecting candidates for genotyping, constructing a computational core, designing a phenotyping panel, or managing genetic collections, the method demonstrates that systematic evaluation of prediction uncertainty is broadly applicable across diverse breeding program objectives where resource constraints necessitate strategic allocation decisions.

The PEV expressions derived in this study enable breeding programs to quantify prediction uncertainty for ungenotyped individuals before making genotyping decisions. The method calculates explicit PEV values for each individual based on their relationships to the training population, providing a mathematical measure of how much uncertainty would remain in their predicted breeding values if left ungenotyped. For example, individuals with stronger genetic connections to the training population through genetic relationships will exhibit lower PEV values, indicating higher expected prediction accuracy. Conversely, candidates from novel genetic backgrounds or with weak training population connectivity will show higher PEV values, signaling greater prediction uncertainty that could potentially be reduced through genotyping.

The three simplified cases derived in this study provide practical tools for different breeding scenarios. Case 1 (no fixed effects) simplifies computations when fixed effects are negligible or when working within homogeneous populations, reducing computational burden without sacrificing accuracy. Case 2 (no phenotypic data) establishes the baseline prediction accuracy achievable from relationships alone, particularly useful for pre-phenotyping decisions and understanding the fundamental contribution of pedigree structure to prediction accuracy. Case 3 (weak phenotypic information) identifies scenarios where phenotypic data collection provides minimal improvement, particularly relevant for low-heritability traits or when training individuals have limited phenotypic records. Together, these cases enable rapid evaluation of prediction uncertainty across different data availability scenarios, helping breeders make informed decisions about resource allocation between genotyping and phenotyping strategies while maintaining computational efficiency when evaluating large numbers of selection candidates.

The reliability of PEV calculations depends mainly on the quality and representativeness of the training population data. Well-established programs with extensive training populations spanning multiple generations provide comprehensive phenotypic and genomic data, enabling accurate variance component estimation that underlies reliable PEV calculations (Pszczola et al., 2012; Misztal et al., 2014). Programs with limited training data or poor variance component estimates may experience reduced accuracy in PEV calculations, potentially leading to suboptimal inferences about prediction uncertainty (Clark et al., 2012). The method accommodates complex population structures and diverse relationship matrix configurations, but the accuracy of the resulting PEV estimates remains fundamentally dependent on the underlying genetic and statistical assumptions being met in practice.

Ultimately, the success of a breeding program depends on its profitability. Genetic progress must be aligned with costs and investments. Integrating this method into an optimization model can enhance the long-term sustainability of genetic evaluation and guide strategic genotyping decisions, directing investments toward areas that deliver the greatest value to breeding programs while considering operational and cost constraints.

### 3.2 Computational considerations

The computational requirements for evaluating the derived PEV expressions are dominated by operations involving the genomic relationship matrix, which is dense and computationally demanding, unlike the sparse design matrices X and Z that characterize fixed effects and phenotypic data structure (Junqueira et al., 2022). The core computational bottleneck lies in calculating the Schur complement $G^{yy}-G^{yt}B^{22}G^{ty}$ , where the dense genomic blocks require $O\left(n_{t}^{3}+n_{y}n_{t}^{2}+n_{y}^{2}n_{t}\right)$ operations per evaluation. While design matrix operations benefit from sparsity and can be computed efficiently, the genomic relationship matrix operations cannot exploit such structural advantages (Misztal et al., 2015).

Matrix decomposition techniques offer computational advantages by exploiting the structure of the genetic covariance matrix and avoiding repeated expensive matrix operations (Strandén and Garrick, 2009; Misztal et al., 2015; Misztal, 2016). For large-scale applications, approximate methods that further exploit matrix structure are required for computational tractability. Two primary approaches can address these challenges: dimensionality reduction and hierarchical approximation.

A common dimensionality-reduction strategy is to approximate the genomic relationship matrix using a principal component (eigen) decomposition (Akdemir et al., 2015; Ødegård et al., 2018), retaining only the leading components that capture most of the variance. This low-rank approximation can then substitute the full matrix, substantially reducing computational cost while preserving the majority of the information content.

Another approach to reduce computational complexity in PEV calculations involves the Algorithm for Proven and Young (APY) methodology (Misztal et al., 2015). APY maintains a core subset of the most informative training individuals while approximating relationships for the remaining population, thereby reducing matrix dimensions in the genetic covariance structure without substantial loss of information. The selection of the core population is a critical step that directly affects the quality of the approximation. Several strategies have been proposed for core selection, including random sampling (Misztal et al., 2015), selection based on maximizing genetic diversity using algorithms such as those described by Pocrnic et al. (2016), and choosing individuals that maximize the number of independent chromosome segments represented in the core (Pocrnic et al., 2016). The optimal core size is related to the effective population size $\left(N_{e}\right)$ of the breed or population, with the number of independent chromosome segments approximated as $4N_{e}L$ , where L is the genome length in Morgans (Strandberg, 2006; Pocrnic et al., 2016). In practice, random selection of core animals has been shown to perform well when the core size is sufficiently large relative to the number of independent chromosome segments (Misztal et al., 2015; Bradford et al., 2017). For the PEV framework presented here, the core population would ideally be selected from the training set to maximize representation of the genetic diversity present in the breeding population, ensuring that the approximated relationship matrix adequately captures the covariance structure between training and young individuals. When applied to PEV derivations, APY enables the decomposition of the relationship matrix into a computationally manageable core component and an approximated remainder. This hierarchical matrix structure allows the Schur complement calculations central to PEV estimation to operate on reduced dimensions. The APY core captures the primary genetic relationships within the training population, while the PEV expressions evaluate prediction uncertainty for young candidates based on their connections to this genetic foundation rather than the full training matrix.

### 3.3 Limitations and assumptions

The prediction error variance method developed in this study relies on several key assumptions that, when violated, may compromise the accuracy and reliability of the derived expressions. Understanding these limitations is important for the appropriate application of the methodology in breeding programs.

#### 3.3.1 Variance component estimation

The PEV calculations assume that variance components are known with certainty. In practice, variance components are estimated from data with associated sampling uncertainty (Junqueira et al., 2017), using either frequentist or Bayesian methods. Misspecification of variance components (Junqueira et al., 2022) directly propagates through the PEV expressions (Meyer, 1989), potentially leading to systematic over- or underestimation of prediction uncertainty. Consequently, theoretical PEV values and realized prediction errors may diverge substantially when variance component estimates are inaccurate. The method presented in this study does not account for this estimation uncertainty, treating variance components as known quantities.

#### 3.3.2 Relationship matrix accuracy

The derivations assume that the relationship matrix G accurately captures genetic covariances among individuals. However, both pedigree-based and genomic relationship matrices are subject to errors. Pedigree errors, including misidentified parentage, unknown parents, or incomplete genealogies, directly distort the covariance structure (Junqueira et al., 2017) and therefore affect PEV calculations. For genomic relationship matrices, genotyping errors, poor marker coverage, or inadequate representation of causal variants can lead to misestimation of realized relationships (Christensen and Lund, 2010). Single-step methods that combine pedigree and genomic information inherit errors from both sources. Since PEV calculations depend fundamentally on the accuracy of genetic covariances between training and young populations through the term $G^{yt}B^{22}G^{ty}$ , relationship matrix errors can impact the reliability of prediction uncertainty estimates.

#### 3.3.3 Model specifications

The derivations of this study explicitly model only additive effects. Although non-additive genetic effects (i.e., dominance and epistasis), are not captured in the presented derivation, this approach can be extended to incorporate non-additive relationship matrices using the same Schur complement logic. For traits where non-additive effects contribute substantially to genetic variance, the PEV expressions will underestimate total prediction uncertainty by failing to account for these unmodeled variance components (Vitezica et al., 2013). The magnitude of this underestimation depends on the proportion of genetic variance attributable to non-additive effects and the extent to which non-additive relationships between training and ungenotyped individuals differ from additive patterns (Muñoz et al., 2014). While single-step genomic BLUP can accommodate dominance or epistatic relationship matrices (Vitezica et al., 2017; Varona et al., 2018), implementing such extensions requires constructing appropriate non-additive relationship matrices and partitioning their contributions within the mixed model equations. Breeding programs selecting for traits with substantial non-additive genetic effect, such as fitness-related traits in livestock or heterosis-dependent traits in crops, should recognize that additive-only PEV calculations may provide optimistic estimates of prediction accuracy.

#### 3.3.4 Absence of selection bias

When relationship matrices incorporate properly defined founder groups or genetic groups to account for population stratification and temporal trends in genetic merit, many systematic biases from historical selection can be mitigated (Misztal et al., 2013; Junqueira et al., 2020). However, within-cohort selection effects and reductions in genetic variance due to linkage disequilibrium (Bulmer effect) remain unaccounted for in standard relationship matrix formulations (Bulmer, 1971). Additionally, when young selection candidates experience substantially different selection intensities or breeding objectives than the training population, the genetic covariances in $G^{ty}$ may not fully represent prediction relationships, potentially affecting PEV estimates (Habier et al., 2007; Patry and Ducrocq, 2011). While the method accommodates genetic groups within the relationship matrix structure, residual selection bias in variance component estimates could still impact PEV calculations in populations under intensive selection.

#### 3.3.5 Static genetic architecture

The derivations assume a constant genetic architecture across environments and over time. Genotype-by-environment interactions (Jarquín et al., 2014), changes in allele effects across generations, or evolution of the genetic background can alter prediction accuracy in ways not captured by static PEV calculations. Breeding programs operating across diverse environments or planning long-term selection strategies should recognize that PEV values calculated under current conditions may not accurately reflect future prediction uncertainty.

## 4 Conclusion

This study presents the equations for computing prediction error variance in ungenotyped individuals under mixed model equations, providing breeding programs with a quantitative foundation for genotyping allocation decisions. The derivation of explicit PEV expressions under different relationship matrix structures enables prospective evaluation of genotyping strategies without requiring actual phenotypic or genomic data ungenotyped individuals. By applying Schur complement methods to partitioned populations, the method accommodates complex breeding program structures while maintaining computational tractability.

The presented mathematical foundation provides the theoretical basis to transition genotyping decisions from experience-based approaches to optimization problems with well-defined objective functions. The PEV expressions reveal how genetic relationships between training populations and ungenotyped individuals directly influence prediction accuracy, providing mathematical indicators to address resource allocation challenges of modern breeding programs where comprehensive genotyping remains economically prohibitive. While implementation requires consideration of computational trade-offs and potential limitations, the framework provides a rigorous mathematical basis for the optimization of limited genotyping resources across diverse plant and animal breeding programs. Future research can build on the presented PEV expressions to implement optimization algorithms and further evaluate the practical benefits and limitations of the approach.

## Data Availability

The original contributions presented in the study are included in the article/supplementary material, further inquiries can be directed to the corresponding author.

## Appendix 1

### Derivation of $B^{22}$

To derive the explicit expression for $B^{22}$ , we apply the block matrix inversion formula to a general 2×2 block matrix following Ouellette (1981).

$$M=\left[\begin{array}{cc} A & B\\ C & D \end{array}\right].$$

Assuming A is nonsingular and $D-{CA}^{-1}B$ is invertible, let $S=D-{CA}^{-1}B$ . The inverse is

$$M^{-1}=\left[\begin{array}{cc} A^{-1}+A^{-1}BS^{-1}CA^{-1} & -A^{-1}BS^{-1}\\ -S^{-1}CA^{-1} & S^{-1} \end{array}\right],$$

so the (2,2) block is

$$M^{22}=S^{-1}={\left(D-{CA}^{-1}B\right)}^{-1}.$$

### Application to the block B

In the main text, the block B (corresponding to $\left(\beta ,u_{t}\right)$ ) is

$$B=\left[\begin{array}{cc} B_{11} & B_{12}\\ B_{21} & B_{22} \end{array}\right]=\left[\begin{array}{cc} X^{⊤}R^{-1}X & X^{⊤}R^{-1}Z_{t}\\ Z_{t}^{⊤}R^{-1}X & Z_{t}^{⊤}R^{-1}Z_{t}+G^{tt} \end{array}\right].$$

$$\begin{array}{ll} B_{11} & =X^{⊤}R^{-1}X\\ B_{12} & =X^{⊤}R^{-1}Z_{t}\\ B_{21} & =Z_{t}^{⊤}R^{-1}X\\ B_{22} & =Z_{t}^{⊤}R^{-1}Z_{t}+G^{tt} \end{array}$$

### Step-by-step derivation

#### Step 1: Compute $B_{11}^{-1}$

$$B_{11}^{-1}={\left(X^{⊤}R^{-1}X\right)}^{-1}.$$

#### Step 2: Compute $B_{21}B_{11}^{-1}B_{12}$

$$B_{21}B_{11}^{-1}B_{12}=Z_{t}^{⊤}R^{-1}X\;{\left(X^{⊤}R^{-1}X\right)}^{-1}\;X^{⊤}R^{-1}Z_{t}$$

#### Step 3: Apply the Schur Complement formula for $B^{22}$

Using the block-inverse formula,

$$B^{22}={\left(B_{22}-B_{21}B_{11}^{-1}B_{12}\right)}^{-1},$$

and substituting the expressions above yields

$$B^{22}={\left(\left(Z_{t}^{⊤}R^{-1}Z_{t}+G^{tt}\right)-Z_{t}^{⊤}R^{-1}X\;{\left(X^{⊤}R^{-1}X\right)}^{-1}\;X^{⊤}R^{-1}Z_{t}\right)}^{-1}$$
